# Protective effects of fenofibrate against acute lung injury induced by intestinal ischemia/reperfusion in mice

**DOI:** 10.1038/srep22044

**Published:** 2016-02-23

**Authors:** Qiankun Zhu, Guizhen He, Jie Wang, Yukang Wang, Wei Chen

**Affiliations:** 1 Department of Parenteral and Enteral Nutrition, Peking Union Medical College Hospital, Peking Union Medical College& Chinese Academy of Medical Sciences, Beijing, 100730, China

## Abstract

This experiment was conducted to evaluate whether pretreatment with fenofibrate could mitigate acute lung injury (ALI) in a mice model of intestinal ischemia/reperfusion (I/R). Male C57BL/6 mice were randomly assigned into three groups (n = 6): sham, intestinal I/R + vehicle, and intestinal I/R + fenofibrate. Intestinal I/R was achieved by clamping the superior mesenteric artery. Fenofibrate (100 mg/kg) or equal volume of vehicle was injected intraperitoneally 60 minutes before the ischemia. At the end of experiment, measurement of pathohistological score, inflammatory mediators and other markers were performed. In addition, a 24-hour survival experiment was conducted in intestinal I/R mice treated with fenofibrate or vehicle. The chief results were as anticipated. Pathohistological evaluation indicated that fenofibrate ameliorated the local intestine damage and distant lung injury. Pretreatment with fenofibrate significantly decreased inflammatory factors in both the intestine and the lung. Consistently, renal creatine levels and hepatic ALT levels were significantly decreased in the fenofibrate group. Moreover, serum systemic inflammatory response indicators were significantly alleviated in the fenofibrate group. In addition, fenofibrate administration significantly improved the survival rate. Collectively, our data indicated that pretreatment with fenofibrate prior to ischemia attenuated intestinal I/R injury and ALI.

Acute lung injury (ALI) is a critically severe, life-threatening condition in ICU, characterized by high mortality of approximately 40%^1^. ALI, along with its more severe form, acute respiratory distress syndrome, often results from a massive insult to the lung, whether be the direct alveolar infiltration or the indirect effects of systemic inflammatory response syndrome or multiple organ dysfunction syndromes^2^. An effective treatment of this life-threatening disorder calls for a better understanding of the molecular pathophysiology of ALI.

The intestine is recognized to be one of the largest immune barrier systems and to be the source of bacteria and endotoxins in the body^3^. Previous reports indicate that the intestinal organ plays a crucial role in the development of ALI^4^. Mesenteric artery ischemia, a commonly seen critical state during small bowel transplantation or trauma, may lead to intestinal ischemia/reperfusion (I/R) injury. During the process of intestinal I/R injury, local bacteria overgrow and translocate, gastrointestinal mobility decreases, and mucosal permeability increases, all of which subsequently lead to the local intestine damage or distant lung injuries through the activation of systemic inflammatory response syndrome or multiple organ dysfunction syndromes^5^,^6^. Intestinal I/R is, therefore, a useful model for the investigation of pathophysiological mechanism of indirect ALI.

Fenofibrate is known as an agonist of the peroxisome proliferator-activated receptor – α (PPARα), the nuclear receptor superfamily member^7^. As a nuclear receptor activator, fenofibrate up-regulates or down-regulates genes that are involved in many pathophysiological processes, such as oxidative stress, inflammation, and leukocyte endothelium interactions^8^,^9^. Recent studies have demonstrated an anti-oxidant, anti-inflammatory, and anti-ischemic role of fenofibrate to attenuate I/R injury in kidney^10^,^11^, liver^12^, brain^13^,^14^ and heart^15^. But to date there are no literatures reporting the protective effects of fenofibrate in I/R injury in the organ of intestine. Moreover, the functional significance of fenofibrate in the intestinal I/R-induced ALI remains unclear. Therefore, this experiment is aimed to investigate whether fenofibrate administration would ameliorate the lung injury and inflammation in the model of intestinal I/R injury.

## Materials and Methods

### Animals

The male C57BL/6 mice (8–10-wk-old; 20 ± 2 g) were purchased from Charles River (Beijing, China). Mice were kept in a temperature (21 ± 2 °C) and humidity (60 ± 5%) controlled room on a 12-hour light/dark cycle. They were fed a standard mice chow and water ad liblitum. All experiments were performed in accordance with the Declaration of Helsinki and were approved by the Institutional Animal Ethical Committee of the Peking Union Medical College Hospital.

### Mice model and drug treatment in mice

Eighteen mice were randomly assigned into three groups (n = 6/group):sham, intestinal I/R + DMSO, and I/R + fenofibrate. Fenofibrate (Abcam, Beijing, China) was suspended with dimethyl sulphoxide (DMSO) solution to the final concentration of 10 mg/ml. The fenofibrate (100 mg/kg of body weight) or equivalent volume of DMSO (vehicle) was intraperitoneally injected into the mice 60 minutes before the ischemia. With respect to the intestinal I/R model, mice were anesthetized with sodium pentobarbital (50 mg/kg, i.p.), allowed to breathe spontaneously during the surgery. After anesthetization, the abdominal wall was opened by midline incision. The superior mesenteric artery was exposed and clamped by an aneurysm clip in order to achieve the intestinal ischemia state when the pulselessness of the mesentery and paleness of the small bowel appeared. On completion of 60 minutes of ischemia, the clip was removed to reperfuse the intestine for 60 minutes. The return of the pulses, the reestablishment of the pink color, and the enhanced intestinal peristalsis were assumed to sign the reperfusion of the intestine. Sham group underwent the identical operation protocols except for the clamping of superior mesenteric artery. The ischemia/reperfusion time was set up according to our experience in our pilot study. All mice were performed a fluid resuscitation with intraperitoneally injection of 1.0 ml of normal saline just after surgery. The mice were sacrificed after completion of intestinal I/R and tissues were quickly harvested. Blood samples were collected by cardiac puncture and centrifuged at 3000 g for 10 minutes in order to collect serum. Tissue and serum were snap frozen in liquid nitrogen and stored at −80 °C until analysis. Additional experiments for observation of survival over the course of 24 hours were performed (n = 15/groups). In the survival experiment, a single dose of 100 mg/kg of fenofibrate or equal volume of DMSO (vehicle) was injected intraperitoneally one hour before the ischemia.

### Histological examination

Morphological changes in the intestine and lungs were examined by hematoxylin and eosin (HE) staining. Every lung lobe was divided equally for histopathological and biochemical investigations. Briefly, portions of left lung (the upper left lobe and lower left lobe) and the right lung (the anterior lobe, the posterior lobe, the median lobe and the post caval lobe) were separately immersed in 10% buffered formalin phosphate, dehydrated in alcohol, embedded in paraffin, and then cut into 4-um sections through the middle of the lobe so that every section included hilum to periphery. The extents of histological injury were evaluated by two blinded experienced investigators. The lung injury was evaluated to the level of absent, mild, moderate, or severe injury (score 0–3) by observing the presence of exudates, hyperemia/congestion, neutrophil infiltration, intra-alveolar hemorrhage/debris and cellular hyperplasia^16^. Ten areas of the lung tissue section for each lung lobe were randomly selected within microscopic high power fields. The lung injury score assessed in each category for every individual mouse was the mean of the scores from the sections of the lungs examined, and total lung injury score was calculated by adding the scores in each of these categories. The intestinal injury was scored on a scale from 0 to 5 as described by Chiu *et al*^17^. Grade 0—Normal mucosal villi. Grade 1—Development of edema in sub-epithelial space, usually at the apex of the villus; often with capillary congestion. Grade 2—Extension of the subepithelial space with moderate lifting of epithelial layer from the lamina propria. Grade 3—Massive epithelial lifting down the sides of villi and a few tips may be denuded. Grade 4—Denuded villi with lamina propria and dilated capillaries exposed and increased cellularity of lamina propria may be noted. Grade 5—Digestion and disintegration of lamina propria and presence of hemorrhage and ulceration. The intestine injury grade in the Chiu scoring system from five sections was averaged to represent the grade for each animal.

### Measurement of organ injury variables

Lung tissues of each lobe from individual mice were removed, rinsed in PBS to remove excess blood. Organs were cut into 1–2 mm pieces and homogenized using a tissue homogenizer.

After two freeze-thaw cycles were performed to break the cell membranes, the homogenates were centrifuged for 5 minutes at 5000 g and the supernatants were used for cytokine determination. Tissue and serum levels of cytokines were determined by enzyme-linked immunosorbent assay (ELISA) kits specific for mouse TNF-α, IL-6, IL-1β NF-κB p65, IκB-α, and HMGB-1 (Abcam, Beijing, China) according to the manufacture’s instruction. NO, iNOS, ALT and creatine levels were measured by using commercial assay kits (Abcam, Beijing, China). The cytokine levels were measured in triplicate and then averaged.

### Western blotting

Tissues were washed with PBS and then homogenized in lysis buffer. The extracted 50 μg of tissue was fractionated on a Bis-Tris gel and transferred to a 0.2-μm nitrocellulose membrane. It was blocked with 5% BSA in Tris-buffer saline with Tween-20 and incubated with a rabbit polyclonal cleaved caspase-3 (1:80; Abcam, Beijing, China) or a rabbit polyclonal Alanine Transaminase (1:1000; Abcam, Beijing, China) overnight. The membranes were then incubated with horseradish peroxidase-labeled goat anti-rabbit IgG (1:3000; Abcam, Beijing, China). Bands were detected using a chemiluminescent peroxidase substrate (ECL, Amersham, Beijing, China) and exposed on a radiograph film (Kodak XBT-1, Beijing, China).

### Statistical analysis

The SPSS program (SPSS Inc., Chicago, IL, USA) was used for statistical analysis. Data were expressed with means ± SD or percentage. The distribution analysis was conducted by Shapiro-Wilk test. If the data were conforming to the normal distribution, then the comparison of multiple groups was performed using ANOVA with LSD to compare differences between individual groups; if the data were not conforming to the normal distribution, then the comparison of multiple groups was carried out by Kruskal-Wallis test, and difference between individual groups were compared by Mann-Whitney U test. The survival rate was analyzed using the Kaplan-Meier log-rank test. Statistical significance was set at a 2-tailed *P* value < 0.05.

## Results

### Fenofibrate ameliorates intestine injury after intestinal I/R

**Figure 1:**
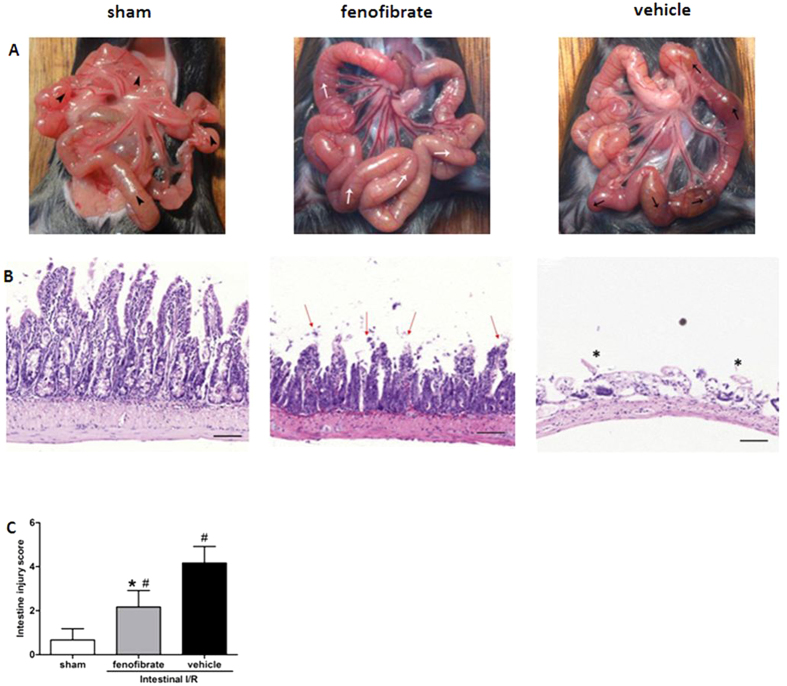
Alteration in gut morphology after I/R. (**A**) Representative images are shown here for the gross morphological alterations of the intestine at the end of the reperfusion. Black arrowheads indicate normal intestine in the sham group. White arrows point to mild I/R injury in the fenofibrate group. Black arrows show severely injured intestine in the vehicle-treated group. (**B**) Histological findings indicate that intestine I/R caused widespread mucosal destruction, loss of villi and infiltration of inflammatory cells, while fenofibrate administration showed protective effects. Red arrows depict mildly injured villi tips, a protective result of fenofibrate. Asterisks indicate severe injury with denuding of villi tips. Original magnification: X500. Scale bars represent 100 μm (**C**) Pathological score of the intestinal injury in the sham, fenofibrate- and vehicle-treated groups after I/R. Data are expressed as mean ±SD (n = 6/group) and compared by Kruskal-Wallis test and Mann-Whitney U test (*P < 0.05 vs. vehicle and ^#^*P* < 0.05 vs. sham).

**Figure 2:**
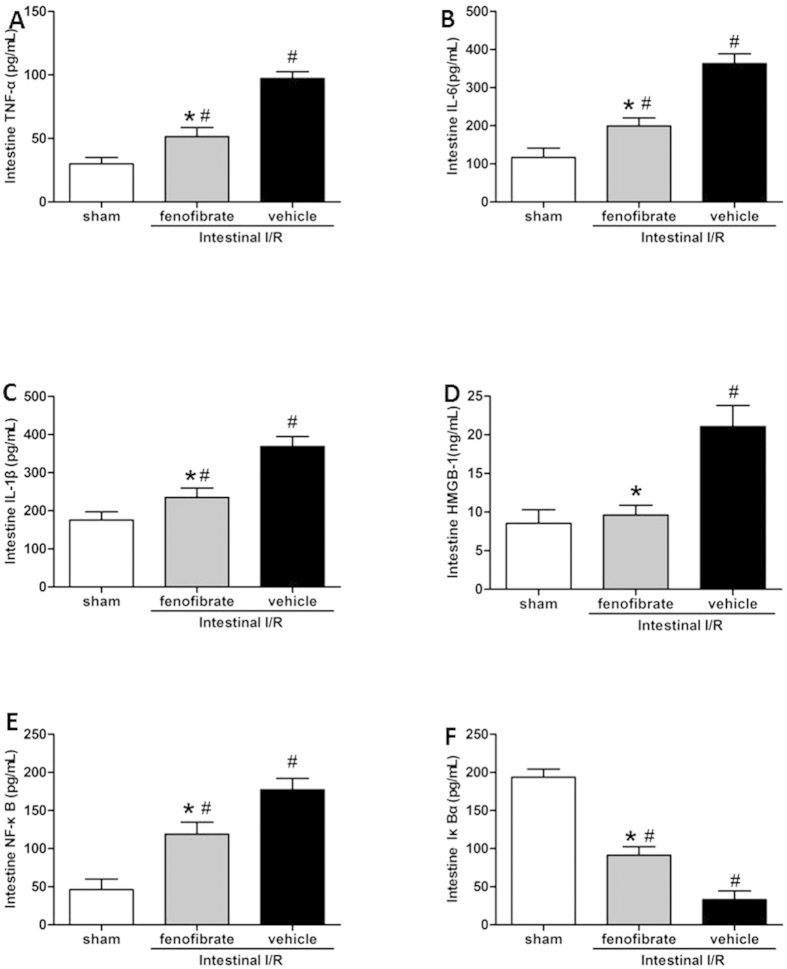
Inflammatory cytokine expression of TNF-α, IL-6, IL-1β, HMGB-1 (**A–D**) and apoptotic variables of NF-κB p65 (**E**) and Iκ Bα (**F**) in the small intestinal tissue of mice of the sham, fenofibrate and vehicle groups (n = 6/group). Cytokines were measured by ELISA and the values were normalized to total cellular protein. Bar graphs shows the mean ±SD. *P < 0.05 vs. vehicle and ^#^*P *< 0.05 vs. sham by one-way ANOVA and LSD test.

After one hour of reperfusion, intestinal I/R led to severe intestine damage characterized by extensive edema, inflammation and necrosis with vascular congestion at the macroscopic appearance compared with that of the sham group. Administration of fenofibrate one hour before the ischemia attenuated the severity of intestinal injury with an ameliorated sign of ischemic darkness and necrosis, although the intestine still had the appearance of moderate edema. (Fig. 1A). In the histopathological examinations of the intestinal walls, the denudation of villi accompanied by severe injury was observed in the gut tissue in the vehicle group compared with the sham group (Fig. 1B). Meanwhile, in the fenofibrate-treated group, the integrity of the intestinal structure and the height of the villi were to some extent protected from the I/R injury in comparison with the vehicle groups (Fig. 1B). Pathological score of the intestine by HE staining indicated that in the fenofibrate-treated group the histological injury score of the intestine was significantly decreased compared with the vehicle group, suggesting the protective effect of fenofibrate in the intestinal I/R injury (Fig. 1C). The inflammatory cytokines including TNF-α, IL-1β, IL-6, and HMGB-1 of the small intestine were significantly reduced in the fenofibrate group compared with the vehicle group (Fig. 2). In terms of the apoptosis indicators, the fenofibrate treated mice were presented with significantly lesser expression level of NF-κB p65 in the small intestine compared with the vehicle treated mice after intestinal I/R. Moreover, Iκ Bα expression in the small intestine of the mice was significantly elevated in the fenofibrate group in comparison with the vehicle group (Fig. 2).

### Administration of fenofibrate suppresses serum variables of multiple organ injury and systemic inflammatory responses after intestinal I/R

It is widely acknowledged that intestinal I/R injury can cause multiple organ injury and systemic inflammatory responses^18^. Pro-inflammatory cytokines are the contributors in the remote organ injury after intestinal I/R. Our hypothesis is based on the assumption that fenofibrate administration can decrease the expression level of inflammatory cytokines in the serum and therefore reduce distant organ injury. So we evaluated the secretion of TNF-α, IL-1β, IL-6 and ALT in the serum after intestinal I/R. Consistent with our expectation, the serum levels of these inflammatory cytokines were significantly decreased compared with the vehicle group (Table 1). Systemic inflammatory indicators TNF-α, IL-1β, IL-6 were significantly reduced in the fenofibrate group by 68.8%, 47.5% and 65.4%, respectively, compared with the vehicle group. The blood markers of remote organ injury ALT was decreased by 29.7% in the fenofibrate group compared with the vehicle group (Table 1).

### Fenofibrate alleviates ALI after intestinal I/R and abates distant organ injuries in the liver and kidney

**Figure 3:**
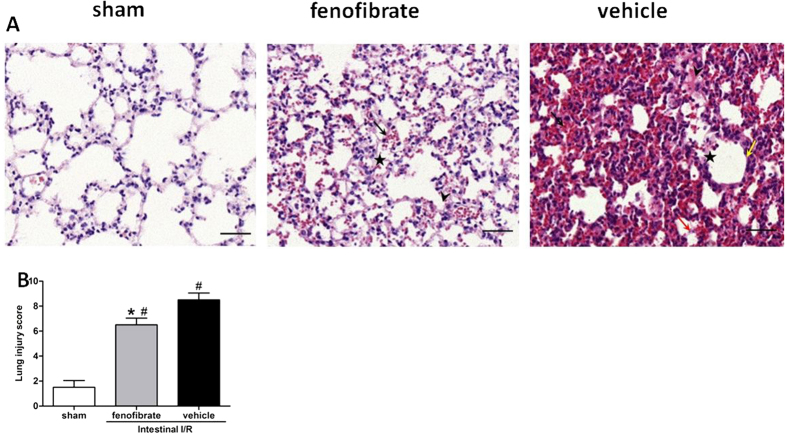
Histological evaluations of the lung after intestinal I/R. (**A**) Representative photomicrography of the lung section. Asterisks indicate the alveolar exudation. Arrowheads depict the edema. Black arrows show the hemorrhagic area. Red arrows represent intra-alveolar hemorrhage/debris. Yellow arrows show cellular hyperplasia. (HE staining; original magnification X200; Scale bars represent 100 μm); (**B**) Lung tissue injury in mice submitted to sham, vehicle and fenofibrate-treated groups. The data represent the mean ± SD (n = 6/group). *P < 0.05 vs. vehicle and ^#^*P *< 0.05 vs. sham by Kruskal-Wallis test and Mann-Whitney U test.

**Figure 4:**
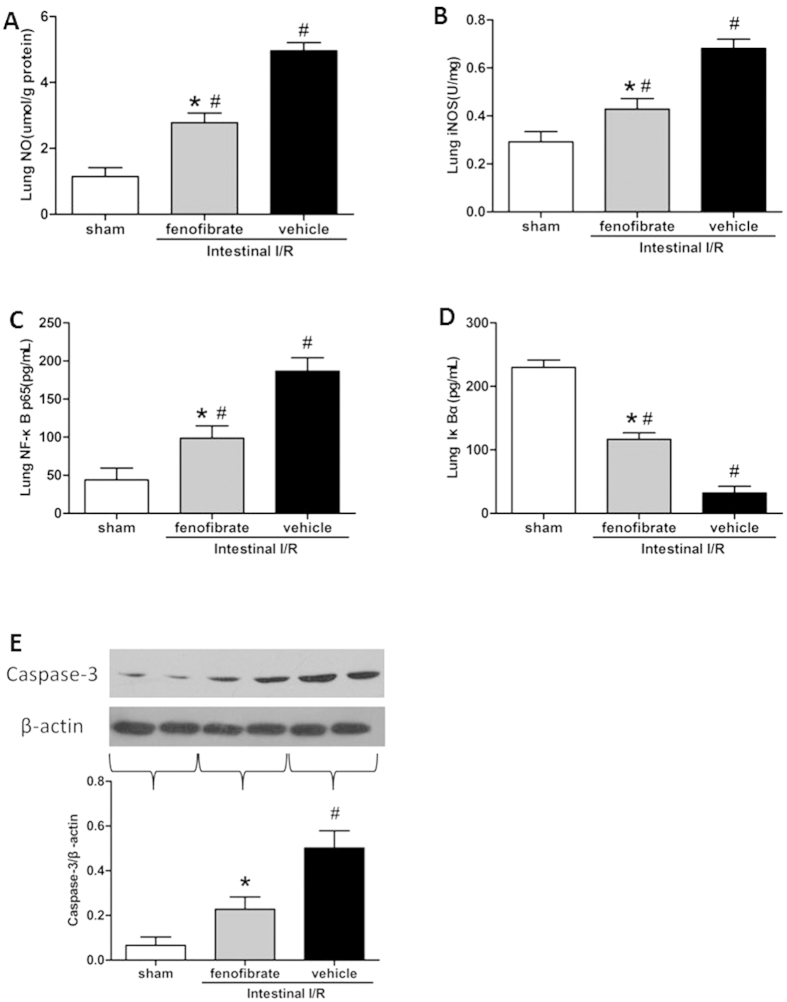
Alterations of lung injury variables after intestinal I/R. (**A**) NO, (**B**) iNOS, (**C**) NF-κB p65, (**D**) Iκ Bα were measured by ELISA in the sham, fenofibrate and vehicle groups; (**E**) Caspase-3 expressions of the lung tissues were assessed by the western blot: results representative of six to ten separate experiments are shown and β-actin is used as internal control. Data are expressed as mean ± SD (n = 6/group) and compared by one-way ANOVA and LSD test (*P < 0.05 vs. vehicle and ^#^*P *< 0.05 vs. sham).

One of the most severely damaged distant organs affected by intestinal I/R injury serves to be the lung^19^. HE staining indicated that lung tissues from vehicle-treated mice were presented with severe histological changes, including intracellular hemorrhage, alveolar congestion, exudates and infiltration of inflammatory cells, in comparison with sham groups (Fig. 3A). However, administration with fenofibrate dramatically ameliorated these ALI histopathological alterations, as seen in the significantly reduced lung injury score (Fig. 3B; Table 2). Apart from the histological indicators, we also measured biochemical variables. These variables included NO, iNOS and Iκ Bα and NF-κB p65 of the lung tissue. The NO and iNOS of the lung tissues were significantly abated in the fenofibrate group compared with the vehicle group, implicating the protective role of fenofibrate of the ALI after intestinal I/R (Fig. 4A,B). Besides, the lung tissue expression of NF-κB p65 was significantly suppressed in the fenofibrate treated group and Iκ Bα was significantly elevated in the fenofibrate group in comparison with the vehicle group (Fig. 4C,D). In order to evaluate the apoptosis in the lungs after fenofibrate treatment, we assessed the caspase-3 expression of the lung tissue. The pulmonary expression level of caspase-3 was significantly up-regulated in the vehicle group after intestinal I/R, while fenofibrate administration significantly decreased the expression level of caspase-3 compared to the vehicle groups (Fig. 4E).

**Figure 5:**
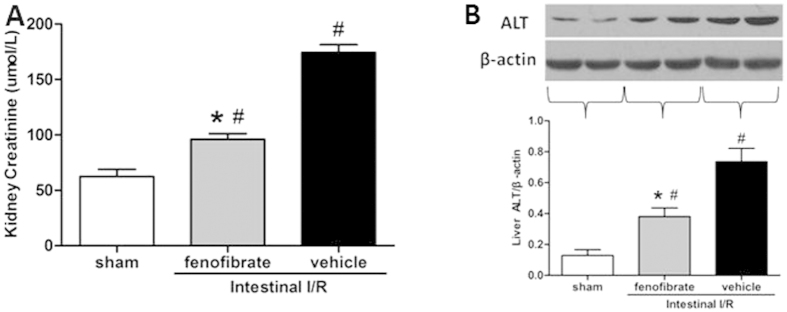
Fenofibrate decreased the distant organ injury after intestinal I/R. (**A**) Renal creatine levels were measured by ELISA. (**B**)Liver tissues were collected and the ALT expressions were evaluated by western blot. Data are expressed as mean ± SD and compared by one-way ANOVA and LSD test (*P < 0.05 vs. vehicle and ^#^*P *< 0.05 vs. sham).

Similarly, in the kidney, the fenofibrate treatment significantly reduced the level of creatine by 44.9% compared to the vehicle group (Fig. 5A). And in the liver tissue, western blot indicated that fenofibrate administration significantly down-regulated the expression of ALT after intestinal I/R when compared with the vehicle group (Fig. 5B).

### Administration of fenofibrate improves the survival rate of mice suffered from intestinal I/R injury

**Figure 6:**
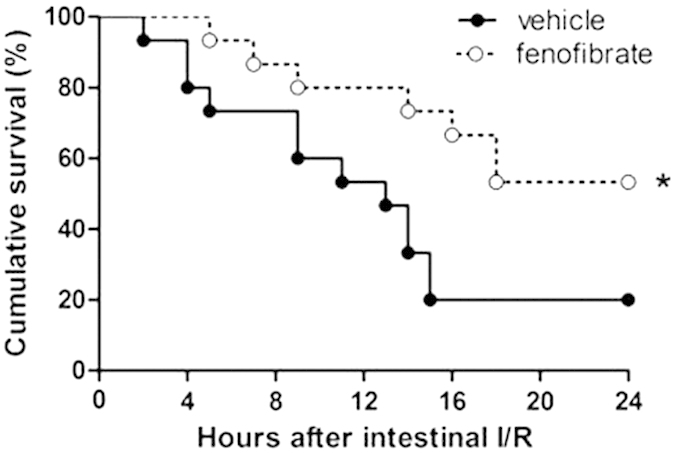
Survival analysis of the fenofibrate and vehicle treated mice after intestinal I/R. Mice subjected to a single dose of fenofibrate (i.p. 100 mg/kg) or vehicle (DMSO) one hour before the ischemia were observed for 24 hours. Each point in the figure represents the mean survival rate at each time point. The survival rate is compared by Kaplan-Meier method and the log-rank test (n = 15/group, *P < 0.05 vs. vehicle).

Since the above results demonstrated the protective effects of fenofibrate, we therefore performed another survival experiment. Mice receiving fenofibrate intraperitoneally 60 minutes before the ischemia were compared with the control mice receiving vehicles. By observing for 24 hours, we compared the survival rate using Kaplan-Meier method and the log-rank test. Seven out of the 15 mice injected with fenofibrate were dead 24 hours post-intestinal I/R, while three of the 15 mice in the vehicle group were still alive 24 hours after intestinal I/R. In general, the survival rate in the fenofibrate group was significantly higher than that of the vehicle group (Fig. 6).

## Discussion

In the present study, we demonstrated that the intestinal I/R negatively affected lung morphology and induced acute lung injury, characterized by systemic inflammation and apoptotic cell death in the lung epithelium. Mice treated with fenofibrate showed an ameliorated inflammatory responses and decreased apoptotic indicators in both the intestine and the distant organ, including the lung, after intestinal I/R. Moreover, fenofibrate improved the survival rate in the model of intestinal I/R.

To the best of our knowledge, this is the first study showing a protective effect of fenofibrate on intestinal I/R-induced ALI, albeit the protective effectiveness has been proved on alleviating the injury in other organs that had undergone I/R^10^,^11^,^12^,^15^. In a rat model of 60-min renal ischemia followed by 24 hours of reperfusion, administration of fenofibrate alleviated the MDA content, the MPO activity, and the generation of proinflammatory mediators. In addition, the expression of nitric oxide (NO), a marker of oxidative damage, was reduced by the treatment of fenofibrate in the renal I/R model^10^. In the cardiac I/R model, fenofibrate effectively attenuated the cardiac injury by reduction of the infarct size and oxidative stress^15^. Our apriori hypothesis was partly based on the anti-oxidant and anti-inflammation properties of fenofibrate. One experiment^20^ with fenofibrate intervention in rheumatoid arthritis patients indicated that a three month long administration of fenofibrate (145 mg/kg) alleviated the inflammatory mediators, C reactive protein and IL-6, in the circulation. Jia, Z *et al*^21^. found out that fenofibrate decreased the level of HMGB1, a downstream protein of TLR4, and therefore attenuated the inflammation in the cardiac cells. Their result on HMGB1 is consistent with one of our experiment results in which the HMGB1 of the small intestine was significantly down-regulated after fenofibrate treatment.

ALI is a commonly seen clinical syndrome of respiratory failure. The pathophysiology mechanism of ALI induced by intestinal I/R remains to be complicated. But it is now widely acknowledged that the ALI is the result of an integrated network of inflammatory mediators and many inflammatory cells^22^. The proinflammatory mediators exert a vital role in the onset of ALI. With regard to the ALI with the etiology of I/R, it has been suggested that occlusion of the super-mesenteric artery causes local intestinal injury and the reperfusion process activates a series of systemic inflammatory responses, which can further damage multiple organs, including the lung, through blood circulations^23^,^24^. In the ALI, the alveolar exudates, bleeding and neutrophils are accumulated in the lung tissue. TNF-α, IL-1β and IL-6 activity is often used to assess the extent of inflammation in intestinal I/R, and there is ample evidence that TNF-α, IL-1β and IL-6 are pivotal inflammatory mediators in the intestinal I/R-caused ALI^25^. In this study, we found that after the administration of fenofibrate the TNF-α, IL-1β and IL-6 activity was significantly decreased in both the intestine and the lung tissue after intestinal I/R. Injured lung epithelium and endothelium also contributes to the development of ALI^26^,^27^, and one feature of this process is the loss of cells through apoptosis. Apoptosis is the major event by which cell death occurs in intestinal I/R mice epithelium and endothelium^28^. To confirm the hypothesis that fenofibrate is involved in regulating apoptosis in the model of intestinal I/R, the caspase-3 activity was used as an apoptotic index to evaluate the degree of apoptosis in the lung tissue. In our experiment, the caspase-3 was significantly reduced in the lung tissue after the fenofibrate treatment. Therefore, we could to some extent state that fenofibrate attenuated I/R-caused ALI in part by apoptosis.

Oxidative stress is another facet of the mechanism of I/R injury. To confirm the effectiveness of fenofibrate in the model of I/R injury, we applied the NO as the oxidative marker which is closely related to I/R^29^. In physiological conditions, NO inhibits the neutrophil release and the platelet accumulation. But in I/R state, NO in the lung tissue is dramatically increased, leading to mitochondria inhibition and energy metabolism dysfunction^30^. The reduced NO concentration in the lung tissue of the fenofibrate treated group is consistent with the ameliorated morphologic presentations of the lung tissue. Therefore, the beneficial effect of fenofibrate in I/R-caused ALI can be partly attributed to its anti-oxidative property. NF-κB p65 is a widely-acknowledged transcriptional factor regulating the distinct inflammatory cytokines in I/R^20^. In normal state, the NF-κB p65 is combined with its inhibitor Iκ Bα within the cytoplasm in an inactive form. When being stimulated under pathological conditions, Iκ Bα becomes phosphorylated and ubiquitinated, and then NF-κB p65 is released and aggregated into the nucleus, activating the related genes to transcript^31^. We demonstrated that fenofibrate markedly inhibited the activation of the NF-κB p65 pathway in the lungs and intestines of mice during intestinal I/R injury, while the Iκ Bα activity was up-regulated compared to the vehicle group, unveiling that the anti-inflammatory property of fenofibrate in the intestinal I/R injury is through an NF-κB p65 dependent pathway. Since the NF-κB p65 activation is reported to be associated with alveolar macrophage activity which is the major source of inflammatory cytokines^4^, the NF-κB p65 pathway in the macrophage seems a reasonable explanation for the mechanism of anti-inflammation of fenofibrate in the protection against ALI in I/R.

There are some limitations of this study. In the first place, because of the amount of histological tissue and serum in mice, we did not access more inflammatory cytokines and oxidative proteins that may have potential functions in it. High throughput ELISA techniques that can measure more proteins with less tissue may be the best solution for this problem in the future. Secondly, although the 24-hour survival analysis demonstrated that pretreatment with fenofibrate improved survival rate significantly, we did not perform any life-sustaining operations during the 24 hours, which are routinely conducted in clinical resuscitations. In clinics, intensive care managements have to be performed in the life-threatening states. Therefore, whether the fenofibrate treatment could attenuate mortality caused by intestinal I/R in clinics is questionable and needs more transitional research to support. But we confidently believe that fenofibrate serve as a bridge between severe intestinal I/R and surgery. In the third place, as only caspase-3 was utilized to assess the apoptosis in the lung tissue, whether fenofibrate has an anti-apoptotic effect during ALI needs to be further investigated. However, it has been reported that TNF-α can trigger the apoptotic cascade by the death receptor/caspase pathway^32^,^33^. Since we observed that fenofibrate decreased TNF-α production, it is sound to infer that fenofibrate alleviates apoptosis in ALI, at least in part, through an indirect manner.

In conclusion, this study indicated that fenofibrate significantly attenuated intestinal I/R injury and prevented the lung from ALI caused by intestinal I/R through the modulation of inflammatory response and apoptosis via NF-κB p65 signaling pathway. On the basis of the present results, fenofibrate seems to be a promising therapeutic agent for the treatment of ALI caused by intestinal I/R.

## Additional Information

**How to cite this article**: Zhu, Q. *et al.* Protective effects of fenofibrate against acute lung injury induced by intestinal ischemia/reperfusion in mice. *Sci. Rep.* **6**, 22044; doi: 10.1038/srep22044 (2016).

## Figures and Tables

**Table 1:** Suppression of serum cytokines by fenofibrate administration after intestinal I/R.

Serum levels	unit	sham	Ischemia/reperfusion	
			fenofibrate	vehicle
TNF-α	*pg/ml *	4.3 ± 1.1	7.9 ± 1.8*^#^	25.4 ± 1.9^#^
IL-1β		12.2 ± 3.3	40.0 ± 3.6*^#^	96.1 ± 3.5^#^
IL-6		25.3 ± 4.0	33.9 ± 4.3*^#^	98.0 ± 3.7^#^
ALT	*U/L*	21.2 ± 4.8	51.2 ± 5.7*^#^	72.8 ± 8.6^#^

To induce intestinal I/R injury, the superior mesenteric artery was blocked for one hour, followed by one hour reperfusion. Serum cytokines of TNF-α, IL-1β, IL-6 and alanine aminotransferase in the sham, fenofibrate-treated and vehicle-treated groups were measured by ELISA, respectively. Data are expressed as mean ± SD (n = 6/group). *P < 0.05 vs. vehicle and ^#^*P* < 0.05 vs. sham.

**Table 2:** ALI score of the lung tissue section after intestinal I/R.

Median (min-max)	exudates	hyperemia/congestion	neutrophil infiltration	intra-alveolar hemorrhage/debris	cellular hyperplasia	Total score
sham	0 (0-1)	1 (0–1)	0 (0–1)	0 (0–1)	0 (0–1)	1.5 (1–2)
fenofibrate	1 (1–2)	1 (1–2)	1 (1–2)	2 (1–3)	1 (0–2)	6.5 (6–7) *^#^
vehicle	2 (1–3)	2 (1–2)	2 (1–3)	1 (1–2)	2 (1–3)	8.5 (8–9)^#^

Pathological evaluations by two blinded experienced investigators of the lung injury in the sham, fenofibrate and vehicle treated groups. The median, minimum and maximum values are shown in the table (n = 6/group), and the total score was calculated by adding the scores in each of these categories. *P < 0.05 vs. vehicle and ^#^*P *< 0.05 vs. sham by Kruskal-Wallis test and Mann-Whitney U test.
